# SEVERE COMPLICATIONS OF BUTTON BATTERY INGESTION IN CHILDREN

**DOI:** 10.1590/S0004-2803.24612026-037

**Published:** 2026-09-28

**Authors:** Vitoria P. MARÓSTICA, Karyne Sumico de Lima Uyeno JORDÃO, Elizete Aparecida LOMAZI, Joaquim Murray Bustorff SILVA, Andrea de Melo Alexandre FRAGA, Maria Ângela BELLOMO-BRANDÃO

**Affiliations:** 1 Pontíficia Universidade Católica de Campinas, Departamento de Pediatria, Faculdade de Ciências Médicas, Universidade Estadual de Campinas, Campinas, SP, Brasil.; 2 Universidade Estadual de Campinas, Faculdade de Ciências Médicas, Departamento de Pediatria, Campinas, SP, Brasil.

**Keywords:** Button-Cell Batteries, child, foreign bodies, COVID-19, Pilhas de Botão, criança, corpos estranhos, COVID-19

## Abstract

**Background:**

Button battery (BB) ingestion has emerged as a global health concern, particularly among children.

**Objective:**

To investigate the clinical outcomes and complications related to BB ingestion among pediatric patients managed at a referral center.

**Methods:**

A case series study was conducted between March 2016 and December 2023, including patients under 14 years old diagnosed with BB ingestion. Clinical and radiological evaluations were performed, followed by endoscopic assessment and treatment when required. Data collected included clinical presentation, endoscopic findings, complications, and outcomes.

**Results:**

A total of 568 cases of foreign body ingestion were initially reviewed, from which only 16 pediatric patients with confirmed BB ingestion were identified between 2016 and 2023. No cases were documented between 2016 and 2018; however, a substantial increase was observed from 2019 through 2022, with a pronounced surge during 2020-2021 that coincided with the COVID-19 pandemic (*P*=0.011). Patient ages ranged from 3 months to 5 years, with a median of 3.22 years (1.62-4.55). Endoscopic retrieval was required in seven children (n=7, 43.7%), whereas nine did not undergo endoscopic intervention owing to the extraesophageal location of the BB. Complications were documented in seven patients, including ulceration (n=6; 37.5%), fistulas (n=3, 18.7%), and stenosis (n=3, 18.7%).

**Conclusion:**

This study highlights the significant risks of severe complications associated with BB ingestion, even in very young children. A significant increase in severe cases referred to our center during the COVID-19 pandemic underscores the importance of awareness, prevention, and timely medical intervention.

## HIGHLIGHTS

• This study evaluated clinical outcomes and complications of button battery ingestion in children at a tertiary referral center.

• A retrospective review (2016-2023) identified 16 pediatric cases among 568 foreign body ingestions.

• Esophageal involvement and complications such as ulcerations and fistulas were frequent.

• These findings highlight the need for prevention, early diagnosis, and prompt specialized management.

## RESUMO

**Contexto:**

A ingestão de bateria tipo botão (BB) tem se tornado um importante problema de saúde pública em todo o mundo, especialmente entre crianças.

**Objetivo:**

Investigar os desfechos clínicos e as complicações relacionadas à ingestão de BB em pacientes pediátricos atendidos em um centro de referência.

**Métodos:**

Foi realizado um estudo de serie de casos retrospectivo entre março de 2016 e dezembro de 2023, incluindo pacientes com menos de 14 anos de idade diagnosticados com ingestão de BB. Todos foram submetidos à avaliação clínica e radiológica, seguida de avaliação endoscópica e tratamento quando indicados. Foram coletados dados referentes à apresentação clínica, achados endoscópicos, complicações e desfechos.

**Resultados:**

Foram revisados 568 casos de ingestão de corpo estranho, dos quais apenas 16 pacientes pediátricos com ingestão confirmada de BB foram identificados entre 2016 e 2023. Nenhum caso foi documentado entre 2016 e 2018; entretanto, observou-se um aumento expressivo a partir de 2019 até 2022, com um crescimento acentuado durante o período de 2020-2021, coincidindo com a pandemia de COVID-19 (*P*=0.011). A idade dos pacientes variou de 3 meses a 5 anos, com mediana de 3,22 anos (1,62-4,55). A remoção endoscópica foi necessária em sete crianças (n=6; 37,5%), enquanto nove não foram submetidas à intervenção endoscópica devido à localização extraesofágica da bateria. Complicações foram documentadas em sete pacientes, incluindo ulceração (n=7; 43,7%), fístulas (n=3; 18,7%) e estenoses (n=3; 18,7%).

**Conclusão:**

Este estudo evidencia o elevado risco de complicações graves associado à ingestão de baterias tipo botão, mesmo em crianças muito pequenas. O aumento significativo de casos graves encaminhados ao nosso centro durante a pandemia de COVID-19 reforça a importância da conscientização, da prevenção e da intervenção médica precoce.

## INTRODUCTION

Button battery (BB) ingestion is a global health concern predominantly affecting children. In recent years, there has been a rapid increase in incidence in the pediatric population, who are at greater risk due to their cohabitation with a growing number of technologies such as electronic toys, remote controls, alarms, cameras, and other devices^1^ ^-^ ^3^.

There is no exact incidence of BB ingestion among children in recent medical literature. Notably, each year more than 3,300 cases are reported to Americans Poisons Controls Centers, including swallowing, inhaling, or lodging in the digestive tract of small, coin-shaped batteries^4^, with a recognized peak incidence among children between six months and 3 years of age^5^. In children, 61.8% of ingested BB originates directly from household products, and approximately 48% were either misplaced or meant for disposal, often in improper disposal practices^6^ ^,^ ^7^.

Reported signs and symptoms of BB ingestion include cough, dysphagia, fever, and vomiting^4^ ^,^ ^8^. BB ingestion requires emergency removal, as it can cause caustic burn injury to its surrounding tissues in a very short period^9^. Hence, if the BB remains in the esophagus for over two hours, it is more likely that complications will arise from the electric current^9^ ^,^ ^10^. Signs and symptoms are often nonspecific in the pediatric population, requiring a high level of suspicion^10^ ^-^ ^15^. Thus, this study evaluates the clinical and endoscopic outcomes of ingested BB in a pediatric population treated in a referenced quaternary hospital.

## METHODS

### Study design and sample

A case series was conducted through a retrospective review of medical records from March 2016 to December 2023. Data was retrieved from the database of the Outpatient Clinic at a tertiary care hospital.

### Data collection

Patients were identified by reviewing the medical records of all children aged ≤14 years with an ICD (International Classification of Diseases) code for foreign body ingestion (ICD-10: T18) who were admitted to the emergency care unit during the study period. Among these cases, patients with BB ingestion were identified and included in the study. Exclusion criteria included patients older than 14 years at the time of BB ingestion and those with loss to follow-up.

The following variables were retrieved for analysis: sex and age at presentation; ingestion episode characteristics, including the number of batteries ingested (single or multiple), whether the ingestion was witnessed or unwitnessed, the setting of ingestion (e.g., at home or under adult supervision), and the time from ingestion to emergency room presentation (in hours). The case period was also recorded and categorized as during the COVID-19 pandemic or the post-pandemic period.

Initial clinical presentation was classified as asymptomatic (incidental finding on X-ray), flu-like symptoms (fever and cough), food refusal and vomiting, or drooling and dysphagia. Imaging data included BB location on X-ray (esophageal or non-esophageal) and whether a computed tomography (CT) scan was performed. Initial management included whether endoscopic removal was performed, which was required for all esophageal cases.

Endoscopic findings and complications included esophageal mucosal injuries, presence of ulcerations, ulcer size greater than 0.5 cm, tracheoesophageal fistula, necrotic esophageal lesions, and hemorrhagic complications. Clinical outcomes included emergency surgical interventions, late complications, duration of nasogastric feeding, and length of hospital stay.

### Statistical analysis and ethical considerations

Descriptive analysis was conducted using frequencies for categorical variables and measures of central tendency and dispersion for continuous variables, which were expressed as mean ± standard deviation and median and interquartile range (IQR; Q1-Q3).

Accident records were retrospectively reviewed and categorized according to two time periods: the pandemic period (2020-2021) and the post-pandemic period (2022-2023). The number of accidents occurring in each period was compared using Fisher’s exact test due to the small number of events observed. Statistical significance was defined as a two-sided p-value <0.05.

The study was approved by the Research Ethics Committee under approval number 5.381.108/2022.

## RESULTS

A total of 568 cases of foreign body ingestion (ICD-10: T18) were initially reviewed between March 2016 and December 2023. Of these, 16 children (11 male and five female) had ingested BBs. One patient ingested two BBs in a single episode, and the other 15 children ingested only one. Our analysis of 16 button battery ingestion cases (Table 1) revealed a male predominance (68.7%) and a median age of 3.22 years (1.62-4.55). The oldest patient was 5 years and 4 months old, whereas the youngest was 3 months and 9 days old. Notably, in the latter case, the battery was reportedly placed in the infant’s mouth by a 3-year-old sibling, highlighting that these events may occur even in very young infants and emphasizing the need for caregiver vigilance and supervision of interactions between young children. The most predominant location where the BB was found was the esophagus, in seven cases.

**TABLE 1:** Demographic and clinical characteristics of button battery ingestion cases (N=16).

Characteristic	Value
Total cases	16 (11 male, 5 female)
Age (median and IQR)	3.22 years (1.62-4.55)
BB ingestion episodes	15 single, 1 double ingestion
Caregivers at admission	14 mothers, 2 fathers
Unwitnessed ingestion	8 (50%)
Home ingestion (supervised)	8 (50%)
Time to ER (n=12)	Mean 13h (1.5-90h)
Pandemic vs post-pandemic	12 (2020-2021) vs 2 (2022-2023)

Initial clinical presentations are shown in Table 2. The clinical course of BB ingestion varied significantly amongst patients. Three children were initially misdiagnosed with flu-like illnesses at primary care centers, with BB ingestion discovered incidentally at routine imaging. Food refusal and vomiting were observed in five cases, while drooling and dysphagia prompted earlier suspicion in others.

**TABLE 2:** Clinical presentation and initial management of button battery ingestion cases (N=16).

Presentation/Intervention	Cases (n=16)
Asymptomatic (incidental X-ray)	3 (18.8%)
Flu-like symptoms (fever, cough)	3 (18.8%)
Food refusal/vomiting	5 (31.3%)
Drooling/dysphagia	5 (31.3%)
BB location (esophagus)	7 (43.8%)
BB location (intestine)	9 (56.2%)
CT scan performed	4 (25%)
Endoscopic removal	7 (all esophageal)
Fasting before admission	9 (56.2%)

Endoscopic management was required for all seven esophageal cases, revealing some sort of mucosal injury. Observed complications (Table 3) primarily included esophageal ulcerations (37.5%), half of which measured <0.5 cm in diameter; this indicated a more favorable prognosis. Two patients developed small tracheoesophageal fistulas, which were detected during the initial endoscopy. Notably, none of the patients required a tracheostomy.

**TABLE 3:** Complications and Outcomes of Button Battery Ingestion Cases (N=16).

Complication/Outcome	Cases (n=16)
Esophageal ulceration	6 (37.5%)
Superficial (<0.5 cm)	2 (12.5%)
Deep (≥0.5 cm)	4 (25.0%)
Tracheoesophageal fistula	2 (12.5%)
Arterioesophageal fistula	1 (6.3%)*
Esophageal dilatation	3 (18.7%)
Surgical ligation/esophagostomy	1 (6.3%)
NG tube feeding (mean duration)	6 days (2 cases)
Hospital stays (endoscopy cases)	10 days (range 1-28)
Readmissions	3 (18.8%)

*Case requiring emergency surgery.

Two children were treated with nasogastric feeding for six days, after which oral feeding was resumed. Both patients developed late substenosis of the proximal esophagus and underwent one esophageal dilatation each.

The most severe case was of a child of 18 months admitted for presenting drooling, coughing, runny nose, and fever. A chest X-ray revealed a BB stuck in their mid-esophagus, which was pushed into the stomach during an emergency endoscopy. The following morning, the battery was removed in a second endoscopy, during which physicians noted a necrotic lesion on the posterolateral wall of the esophagus with light bleeding. A few hours later, the child progressed with profuse, bright red bleeding from the nose and mouth followed by bradycardia, hemodynamic instability, and cardiac arrest that was promptly reversed. An emergency surgical procedure identified a necrotic lesion on the posterior wall of the thoracic esophagus, eroding an anomalous right subclavian artery that was causing the bleeding. This artery was successfully ligated, and due to the extent of the esophageal lesion, esophagostomy and gastrostomy were performed. The patient recovered from these events and was discharged 20 days later. One year later, he was readmitted for esophageal reconstruction and is presently doing well, feeding by mouth and needing eventual esophageal dilatations.

No cases of BB ingestion were reported from 2016 to 2018; two cases were recorded in 2019, three in 2020, nine in 2021, two in 2022, and none in 2023. We found a statistically significant decrease in the number of accidents from 12 during the pandemic period (2020-2021) to 2 during the post-pandemic period (2022-2023) (*P*=0.011).

## DISCUSSION

After literature analysis, the findings of our study are compatible with previous data^1^ ^,^ ^4^ ^,^ ^11^ regarding BB ingestion in children, especially under six years old. A French retrospective descriptive study reported a median age of 28 months (13-62 months) and predominantly male children (84.6%). Additionally, 25 of the 26 BB ingested had a diameter ≥20 mm. Although our median age was higher than in the French study, we found BB ingestion by children as young as three months old. Nevertheless, boys represented most of our cases. Data from the French study reported a mean hospital stay of 15.4 days, higher than our findings, and highlighted that the mean hospital stay cost was €38,751 (range, €5,130- 119,737)^15^.

As demonstrated in previous studies ^4^ ^,^ ^6^ ^,^ ^8^ ^,^ ^11^ ^-^ ^14^, BB ingestion in children often presents with nonspecific signs and symptoms. When the ingestion event is unwitnessed by caregivers and only identified through subsequent diagnostic investigations, delayed recognition may occur.

This delay increases the risk of complications by postponing both detection and appropriate clinical management. These challenges underscore the importance of maintaining a high index of suspicion for BB ingestion in pediatric patients with unexplained symptoms.

A study by the Button Battery Task Force^12^ found 56% of complications and a mortality of 4.8%. Although nearly half of our study’s samples experienced complications, no fatalities were reported. Esophageal ulceration was the most common complication. One patient developed severe hypovolemic shock due to an arterio-esophageal fistula but was promptly treated and survived; in contrast, a similar fatal case was reported in a 14-month-old child who died due to complications from BB ingestion in a Denmark case report^16^. Only a few computed tomography (CT) scans were performed in our case series, highlighting the importance of having a consistent guideline that enforces standardized imaging protocol in these specific cases^19^.

There is a consensus towards conduct when the BB is impacted in the esophagus; on the other hand, it is controversial when it is located downwards in the gastrointestinal tract. A retrospective multicenter pediatric study in North America found gastric tissue injuries in 60% of all children who underwent endoscopic removal of BB in the stomach. Additionally, 54% of initially asymptomatic patients showed some degree of gastric injury, including one case of pneumoperitoneum secondary to gastric perforation^12^ ^,^ ^17^ ^,^ ^18^.

The European Society for Pediatric Gastroenterology Hepatology and Nutrition (ESPGHAN) task force for BB ingestions recommends a seven-day follow-up with radiographic evaluation for asymptomatic patients with batteries located below the esophagus and ingestion occurring within 12 hours, unless caregivers have documented the battery’s passage in the child’s stool^19^. In symptomatic cases, and if the battery remains in the stomach after seven to 14 days, endoscopic removal is recommended. When the battery is lodged in the small intestine, ESPGHAN recommends joint evaluation by a pediatric surgeon. For ingestions occurring over 12 hours prior, endoscopy should be considered to rule out esophageal damage, and computerized tomography scans should be performed to assess vascular damage^20^. In the present study, the main complications occurred when the battery was lodged in the esophagus.

An Indian retrospective study^21^ of 55 cases of BB ingestion reported only 3 complications, a striking contrast to our findings. This discrepancy may stem from differences in healthcare system structures between countries, including variations in access to care, referral patterns, and management protocols for foreign body ingestion. Such factors complicate direct comparisons across studies. Additionally, while our analysis included only 16 cases, our institution serves as a quaternary referral center, likely receiving the most severe presentations. This selection bias may account for the higher proportion of complicated cases in our case series.

Our findings point to a higher number of cases during 2020-2021, coinciding with the COVID-19 pandemic. Data from the US Consumer Product Safety Commission^22^ indicated a 93% increase in emergency room visits related to BB incidents from March to September 2020 compared with the same period in 2019, specifically among children aged 5 to 9. These accidents primarily involved battery ingestion but also included cases of ear or nose insertion. A study conducted in London found an increase in pediatric foreign body ingestions during the COVID-19 pandemic, including BB and magnets, which the authors attributed to the disruption of home and work settings, including a significant reduction in enrollment in daycares and schools worldwide during the coronavirus pandemic^23^.

Our findings point to a higher number of cases during 2020-2021, coinciding with the COVID-19 pandemic. Data from the US Consumer Product Safety Commission^22^ indicated a 93% increase in emergency room visits related to BB incidents from March to September 2020 compared with the same period in 2019, specifically among children aged 5 to 9. These accidents primarily involved battery ingestion but also included cases of ear or nose insertion. A study conducted by Festa et al., 2021^23^ in London also found a 2.5-fold increase in pediatric foreign body ingestions, including BB and magnets, during the pandemic. These findings have been attributed to prolonged home confinement, disruption of school and daycare attendance, and changes in supervision patterns. Additionally, increased exposure to household electronic devices containing BB may have contributed to the higher incidence observed during this period^23^.

The significant reduction in accidents observed after 2021 supports the hypothesis that pandemic-related environmental and behavioral factors contributed to the occurrence of these events. As schools and daycare centers progressively reopened, children likely spent less time in household environments, potentially reducing their exposure to button batteries and other domestic hazards.

To our knowledge, data on BB ingestion in Latin America remain scarce. Most of the available evidence from the region consists of isolated case reports^24^ and studies evaluating foreign body ingestion as a broader category^25^, leaving important gaps in our understanding of the epidemiology, clinical presentation, complications, and outcomes of BB ingestion in Latin American children. Nevertheless, our findings should be interpreted considering several limitations, including the relatively small sample size, retrospective design, and single-center setting. In addition, information regarding the size of the ingested button batteries was not consistently available.

## CONCLUSION

This study highlights the serious complications associated with BB ingestion, which can occur even in very young children, and the significant risks and potential complications resulting from these events. During the COVID-19 pandemic, there was a marked increase in severe BB ingestion cases referred to our tertiary referral center. Additionally, risks can be mitigated through improved safety and management of devices containing BB, along with increased supervision of young children who should not have access to such devices.

## Data Availability

The research data are available in a public repository (Doi: 10.25824/redu/J2ALNW)
